# Supplementary material for: Surface Microbiome Profiling of Dental Elevators Using Third‐Generation Sequencing: Implications for Infection Control in Dental Practice
Source: Microbiologyopen. 2025 Dec 8;14(6):e70178. doi: 10.1002/mbo3.70178 (PMC12685768; doi:10.1002/mbo3.70178)
Supplement: Supplementary file 1 — customer_backup. [file MBO3-14-e70178-s001.zip › customer_backup/customer_backup/function_analysis/COG/allsample/K2species/allsample.reabundance_Class2Class.txt.pdf]

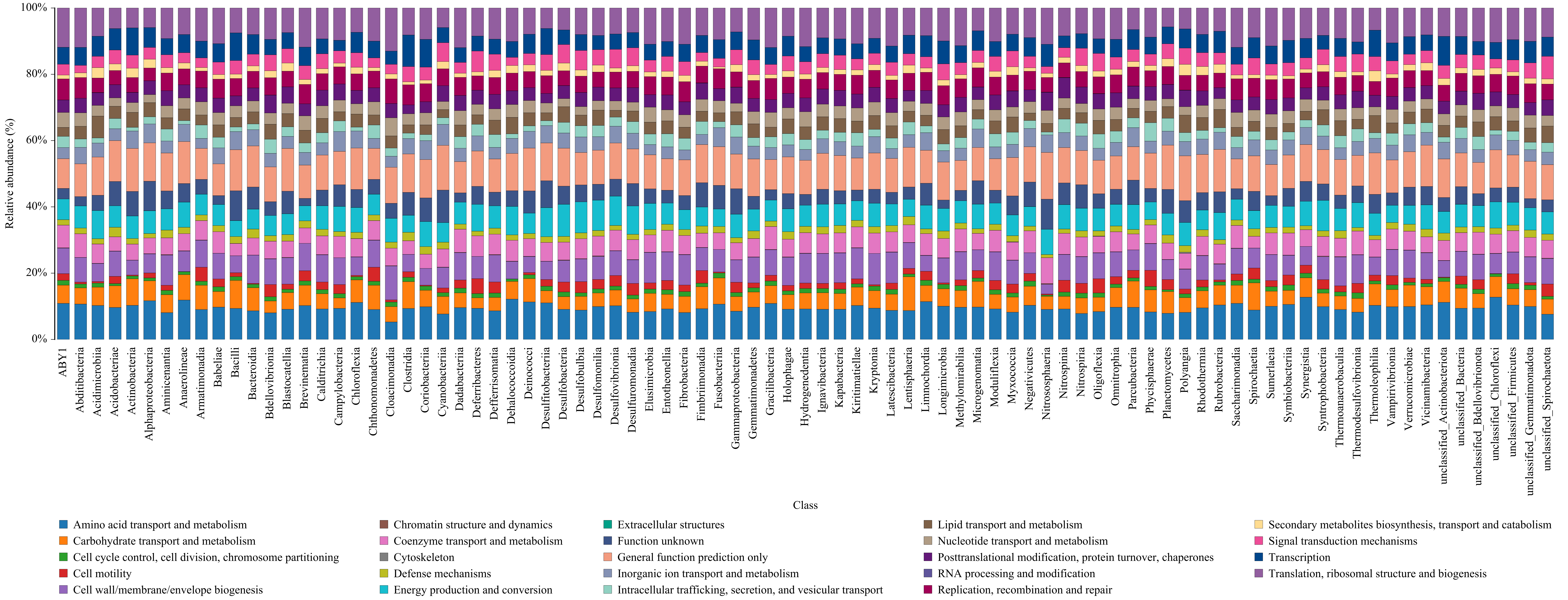
100%
80%
60%
40%
20%
0%
Relative abundance (%)
ABY1
Abditibacteria
Acidimicrobiia
Acidobacteriae
Actinobacteria
Alphaproteobacteria
Aminicenantia
Anaerolineae
Armatimonadia
Babeliae
Bacilli
Bacteroidia
Bdellovibrionia
Blastocatellia
Brevinematia
Calditrichia
Campylobacteria
Chloroflexia
Chthonomonadetes
Cloacimonadia
Clostridia
Coriobacteriia
Cyanobacteriia
Dadabacteriia
Deferribacteres
Defferrisomatia
Dehalococcoidia
Deinococci
Desulfitobacteriia
Desulfobacteria
Desulfobulbia
Desulfomonilia
Desulfovibrionia
Desulfuromonadia
Elusimicrobia
Entotheonellia
Fibrobacteria
Fimbriimonadia
Fusobacteriia
Gammaproteobacteria
Gemmatimonadetes
Gracilibacteria
Holophagae
Hydrogenedentia
Ignavibacteria
Kapabacteria
Kiritimatiellae
Kryptonia
Latescibacteria
Lentisphaeria
Limnochordia
Longimicrobia
Methylomirabilia
Microgenomatia
Moduliflexia
Myxococcia
Negativicutes
Nitrososphaeria
Nitrospinia
Nitrospiria
Oligoflexia
Omnitrophia
Parcubacteria
Phycisphaerae
Planctomycetes
Polyangia
Rhodothermia
Rubrobacteria
Saccharimonadia
Spirochaetia
Sumerlaeia
Symbiobacteriia
Synergistia
Syntrophobacteria
Thermoanaerobaculia
Thermodesulfovibrionia
Thermoleophilia
Vampirivibrionia
Verrucomicrobiae
Vicinamibacteria
unclassified_Actinobacteriota
unclassified_Bacteria
unclassified_Bdellovibrionota
unclassified_Chloroflexi
unclassified_Firmicutes
unclassified_Gemmatimonadota
unclassified_Spirochaetota
Class
Amino acid transport and metabolism
Carbohydrate transport and metabolism
Cell cycle control, cell division, chromosome partitioning
Cell motility
Cell wall/membrane/envelope biogenesis
Chromatin structure and dynamics
Coenzyme transport and metabolism
Cytoskeleton
Defense mechanisms
Energy production and conversion
Extracellular structures
Function unknown
General function prediction only
Inorganic ion transport and metabolism
Intracellular trafficking, secretion, and vesicular transport
Lipid transport and metabolism
Nucleotide transport and metabolism
Posttranslational modification, protein turnover, chaperones
RNA processing and modification
Replication, recombination and repair
Secondary metabolites biosynthesis, transport and catabolism
Signal transduction mechanisms
Transcription
Translation, ribosomal structure and biogenesis
